# Capsaicin ameliorates diabetic retinopathy by inhibiting poldip2-induced oxidative stress

**DOI:** 10.1016/j.redox.2022.102460

**Published:** 2022-09-03

**Authors:** Kou Liu, Xiang Gao, Chengyang Hu, Yanchao Gui, Siyu Gui, Qinyu Ni, Liming Tao, Zhengxuan Jiang

**Affiliations:** a Department of Ophthalmology, The Second Affiliated Hospital, Anhui Medical University, Hefei, Anhui, 230601, China; b Department of Humanistic Medicine, School of Humanistic Medicine, Anhui Medical University, Hefei, Anhui, 230032, China

**Keywords:** Capsaicin, Diabetic retinopathy, Poldip2, Nox4, Oxidative stress

## Abstract

**Background:**

Oxidative stress and the resultant hyperpermeability play a vital role in the pathogenesis of diabetic retinopathy (DR). Poldip2 has been implicated in H_2_O_2_ production, but the effects of capsaicin on poldip2 have not been reported.

**Methods:**

Diabetic Sprague-Dawley (SD) rats induced with STZ were treated with capsaicin or AAV_9_-*poldip2*-shRNA, and human retinal microvascular endothelial cells (HRMECs) were treated with capsaicin or *poldip2* siRNA.

**Results:**

Current data indicated that the expression of PPARγ, poldip2, Nox4, VCAM-1, HIF-1α, and VEGF increased in rat retinas with DR and in HRMECs treated with high glucose. The production of ROS or H_2_O_2_ in the tissues, serum, and cells increased, and the paracellular permeability of cultured HRMECs with high glucose significantly increased. In addition, overt hyperpermeability of retinal microvessels and increased retinal neovascularization in diabetic rats were observed. However, capsaicin treatment inhibited these increases and suppressed the expression of PPARγ by enhancing its phosphorylation and ubiquitination in the retinas of DR rats. Poldip2 knockdown in HRMECs or its silencing in the retina of DR rats concomitantly led to reduced levels of Nox4, VCAM-1, HIF-1α, VEGF, ROS, and H_2_O_2_, and the paracellular permeability of HRMECs or the hyperpermeability of retinal microvessels in diabetic rat retinas decreased. Similarly, after PPARγ knockdown in HRMECs, poldip2, Nox4, HIF-1α, VEGF, ROS, and H_2_O_2_ decreased, and the monolayer paracellular permeability was reduced accordingly.

**Conclusion:**

Capsaicin may ameliorate diabetic retinopathy by activating TRPV1 and suppressing the PPARγ-poldip2-Nox4 pathway.

## 1 Introduction

Diabetic retinopathy (DR), a microvascular complication of diabetes, is the leading cause of diabetes-associated visual damage or loss among working-age adults worldwide [1]. It is estimated that the number of DR patients will rise to 191 million by 2030. As more research has been conducted, our understanding of the pathophysiological mechanisms of diabetic retinopathy has evolved. The main pathophysiological changes in DR are caused by long-term hyperglycemia. Hyperglycemia can induce oxidative stress and inflammation of retinal microvessels, resulting in thickening of the retinal capillary basement membrane, increasing retinal vascular permeability, and leading to neovascularization [2].

Oxidative stress is caused by an imbalance between free radical formation and scavenging, and superoxide anions (O_2_^·–^) and hydrogen peroxide (H_2_O_2_) are important components of reactive oxygen species (ROS). Oxidative stress is a critical contributor to DR pathogenesis [3]. Under physiological conditions, the primary function of ROS (H_2_O_2_) is to mediate various biological responses as signaling molecules. However, under pathological conditions, excessive accumulation of ROS (H_2_O_2_) can elicit the transcription factor hypoxia-inducible factor-1α (HIF-1α), leading to increased expression of vascular endothelial growth factor (VEGF) and inflammatory mediators, which damage retinal microvessels [4,5]. In diabetes mellitus (DM), ROS (H_2_O_2_) is produced through various pathways, and NADPH oxidase (Nox) becomes the primary source of H_2_O_2_ in the vascular system. In recent years, evidence suggests that Nox1, Nox2, and Nox4 participate in pathological angiogenesis [6]. Nox4 is a major NADPH oxidase of the Nox family in human retinal vascular endothelial cells (HRMECs) and is mainly distributed in the mitochondria and endoplasmic reticulum. One study showed that the expression of Nox4 and VEGF in the retina of db/db mice was increased, and that Nox4 siRNA injection *in vivo* could reduce the expression of Nox4 and VEGF in the retina and decrease retinal vascular permeability [7]. Nox4 could promote retinal neovascularization through ROS-dependent regulation of the VEGF/VEGFR2 signaling pathway and various inflammatory signaling pathways, ultimately contributing to DR [8].

ROS production and hyperglycemia can induce endothelial cells to overexpress vascular cell adhesion molecule-1 (VCAM-1) [9]. VCAM-1 is a member of the immunoglobulin (Ig) family. It can act as a scaffold for leukocyte migration in the endothelium and promotes the adhesion of vascular endothelial cells to leukocytes. Published studies have shown that poldip2 is an important mediator of leukocyte infiltration into the brain. It has also been noted that in rat brain microvascular endothelial cells (RBMECs), silencing poldip2 can weaken the upregulation of VCAM-1 induced by TNF and reduce the adhesion between leukocytes and brain endothelial cells [10].

Polymerase δ-interacting protein 2 (poldip2) is a multifunctional protein that plays a vital role in DNA replication, damage repair, mitochondrial function, and cell migration. Studies have reported that poldip2 is a crucial regulator of endothelial barrier function and the inflammatory response [11,12], and that poldip2 binds to p22phox and enhances Nox4 activity [13]. Overexpression of poldip2 in vascular smooth muscle cells (VSMCs) specifically promotes ROS production by increasing Nox4 activity. In addition, a recent study indicated that poldip2 knockout reduced H_2_O_2_ production in poldip2^+/−^ mice [14]. Our published research revealed that the expression of poldip2 was reduced in hepatocytes under DM, resulting in the deficiency of H_2_O_2_, eventually leading to a disorder of glucose and lipid metabolism in the diabetic liver [15]. This study lays the foundation for exploring the potential role of poldip2 in regulating H_2_O_2_ production in DR.

Our preliminary experiment showed that the expression of poldip2 and Nox4 was significantly increased by high glucose concentrations in HRMECs *in vitro*, accompanied by the elevation of HIF-1α and VEGF. Therefore, we speculated that hyperglycemia might enhance poldip2 expression in retinal microvessels under DM conditions, thus causing retinal microangiopathy of DR by regulating the Nox4-H_2_O_2_–HIF-1α pathway.

Peroxisome proliferator-activated receptor gamma (PPARγ) is a member of the nuclear receptor super-family, acting as transcription factor and playing an important role in glucose and lipid metabolism. It has been documented that the expression of the retinal PPARγ in diabetic rats significantly increased [16]. In addition, study has shown that capsaicin can inhibit the expression of PPARγ [17].

Finding effective prevention and treatment measures has always been a focus of research. Capsaicin (CAP) is an agonist of the transient receptor potential vanillic 1 (TRPV1), the primary active ingredient in chili [18]. It works by activating TRPV1 and has antioxidant, anti-inflammatory, and protective roles in the cardiovascular system [18]. CAP was shown to be an effective angiogenesis inhibitor *in vitro* and *in vivo* [19]. A previous study showed that CAP might reduce apoptosis of diabetic retinal cells by upregulating CGRP [20]. Our preliminary experiments showed that compared with high glucose treatment in HRMECs, CAP treatment significantly activated TRPV1 while markedly inhibiting the expression of poldip2 and Nox4. In the present study, we explored the role of CAP in ameliorating DR by suppressing the poldip2-Nox4-H_2_O_2_ pathway.

## 2 Materials and methods

### 2.1 Reagents and materials

**Table 1:** Contains information regarding the catalogue number of all reagents, cell lines, and kits used.

categories	vendor	catalog number	dilution ratio
Primary antibodies			
TRPV1	abcam	ab6166	1:1000 (WB)
			1:100 (IHC)
PPARγ	santa cruz	sc-7237	1:200 (WB)
p-PPARγ	affinity	AF3284	1:500 (WB)
Poldip2	abcam	ab181841	1:1000 (WB)
			1:100 (IHC)
ubiquitin	proteintech	10201-2-AP	1:1000 (WB)
Nox4	novus	NB110-58849	1:1000 (WB)
			1:100 (IHC)
HIF-1α	abcam	ab1	1:500 (WB)
VCAM-1	abcam	ab134047	1:2000 (WB)
VEGF	abcam	ab32152	1:1000 (WB)
actin	sigma	A5441	1:5000 (WB)
Secondary antibodies			
goat anti-mouse IgG	ZSGB-BIO	ZB-2301	1:5000 (WB)
goat anti-rabbit IgG	ZSGB-BIO	ZB-2305	1:5000 (WB)
**reagents**		**vendor**	**Catalog number**
Total cholesterol assay kit		NanJing JianCheng	A111-1-1
Triglyceride assay kit		NanJing JianCheng	A110-1-1
High-density lipoprotein cholesterol assay kit		NanJing JianCheng	A113-1-1
Low-density lipoprotein cholesterol assay kit		NanJing JianCheng	A112-1-1
BCA protein assay kit		Beyotime	P0010
ROS assay kit		Sigma	D7008
Reactive oxygen species assay kit		Beyotime	S0033S
Hydrogen peroxide assay kit		Beyotime	S0038
Hematoxylin-Eosin kit		Solarbio	G1120
Cell Counting Kit-8 (CCK-8)		Targetmol	C0005
Capsaicin		Sigma	15971
Streptozotocin		Sigma	S0130
Evans Blue		Sigma	E2129
RIPA Lysis Buffer		Beyotime	P0013B
Phenylmethanesulfonyl fluoride		Beyotime	ST505
Phosphatase inhibitor cocktail		Beyotime	P1082
PVDF membrane		Millipore	ISEQ00010
Evans blue dye		Sigma-Aldrich	E2129
Matrigel Matrix		Corning	356234
FAS eye fixative solution		Servicebio	G1109
Antifade mounting medium		Beyotime	P0131
HRMECs		Innoprot	P10880
Endothelial cell medium		ScienCell	1001
Fetal bovine serum (FBS)		Gibco	10099141C
Penicillin-streptomycin		Beyotime	C0222

### 2.2 Diabetic rat modeling

Seventy 8-week-old male SD rats (220 ± 10 g) were obtained from the Laboratory Animal Center of Anhui Medical University, and all animal experimental protocols were approved by the Animal Ethics Committee of Anhui Medical University. The rats were housed under 12 h light/dark cycles, at temperature of 23 ± 2 °C, humidity 50 ± 5%, and with ad libitum access to food and water. The rats were acclimatized for at least 1 week before the experiments. Fifty rats were randomly chosen to be intraperitoneally injected with STZ (dissolved in 0.1 mol/L citrate buffer, pH 4.5) (Table 1) at 60 mg/kg body weight (bw) to induce type 1 diabetes. 72 h after STZ injection, the animals with a fasting blood glucose concentration ≥16.7 mmol/L were considered diabetic [16]. The success rate of the modeling was 86%, with 43 rats being confirmed.

### 2.3 Capsaicin treatment

After 4 weeks of successful modeling, the rats without STZ injection were chosen as the normal control group (NC, n = 10), and the diabetic rats were randomly divided into an untreated diabetic group (DM, n = 10) and a diabetic group with CAP treatment (DM + CAP, n = 10). Rats in the DM + CAP group received 0.5 mg/kg bw/d CAP (dissolved in DMSO) by oral gavage for 8 weeks. Rats in the NC and DM groups received equal volumes of DMSO orally. Body weight and fasting blood glucose levels in the three groups were monitored weekly.

### 2.4 *In vivo* knockdown of poldip2 by AAV_9_-*poldip2*-shRNA

The rats were randomly divided into three groups: the normal control group (NC, n = 10), the diabetic group treated with scrambled-AAV_9_-shRNA (DM + NC-shRNA, n = 10), and the diabetic group treated with AAV_9_-*poldip2*-shRNA (DM + *poldip2-*shRNA, n = 10). After anesthetization with pentobarbital sodium (50 mg/kg), the rats in the DM + *poldip2-*shRNA group were injected with 5 μl AAV_9_-*poldip2*-shRNA in the vitreous cavity, 2 mm posterior to the limbus, using a 33-gauge needle. Rats in the NC group were left untreated, and rats in the DM + NC-shRNA group were injected intravitreally with 5 μl scrambled-AAV_9_-shRNA. The AAV_9_-*poldip2*-shRNA (rat) targeting sequence was 5′-gttcagtttccacatgtttcc-3'. For the scrambled shRNA, the sequence was 5′-cgctgagtacttcgaaatgtc-3'. Tissue samples from rat eyes were collected 4 weeks after virus injection.

### 2.5 Detection of serum TC, TG, HDL, and LDL levels

Whole blood was taken from the abdominal aorta of rats, and the serum was separated by centrifugation at 3000 rpm, 4 °C for 15 min, and stored at −80 °C. The serum concentrations of total cholesterol (TC), triglyceride (TG), high-density lipoprotein cholesterol (HDL), and low-density lipoprotein cholesterol (LDL) were measured using assay kits according to the manufacturer's instructions.

### 2.6 Hematoxylin-eosin (HE) staining

After the rats were anesthetized with pentobarbital sodium (50 mg/kg bw), the eyeballs were obtained and immersed in FAS eye fixative solution for 24 h. The eyeballs were then immersed in 75%, 85%, 90%, and 95% alcohol, anhydrous ethanol, xylene, and melting paraffin for dehydration and wax dipping. Wax-soaked eyeballs were embedded in the paraffin processor (EG1150, Leica, wetzlar, Germany). The cooled wax blocks were sliced to a thickness of 4 μm on a microtome (RM2235, Leica, wetzlar, Germany). The slices were dewaxed with xylene, rehydrated in graded alcohol solutions including anhydrous ethanol, 95%, 85%, and 75% alcohol, then put into distilled water, and stained with hematoxylin solution for 5 min and eosin dye for 5 min. The tissues were then dehydrated in alcohol and xylene, sealed with neutral gum, and observed under a microscope (DM3000, Leica, wetzlar, Germany). The thickness of the retina (ILM + NFL + GCL, IPL, INL, OPL and ONL) was quantified using ImageJ software (the quantification point was 1 mm from the optic disc). The ILM and NFL are relatively thin and difficult to be accurately distinguished from the GCL on the HE-staining section, we actually included the ILM and NFL when quantifying the GCL. The average value was calculated according to 3 left eyes from 3 animals.

### 2.7 Tissue immunofluorescence

Paraffin sections of rat retina were dewaxed with xylene, rehydrated in graded alcohol solutions including anhydrous ethanol, 95%, 85%, and 75% alcohol, blocked with 3% BSA. The antigen was retrieved by incubation at 95 °C in antigen retrieval solution (0.01 M citrate, pH = 6) for 30 min, after which the slices incubated with primary antibodies (anti-TRPV1, anti-Nox4, and anti-poldip2) at 4 °C overnight, and subsequently incubated with fluorescent-dye conjugated secondary antibodies for 2 h. The sections were sealed with an antifade mounting medium and immediately observed under a fluorescence microscope.

### 2.8 Evans blue permeability assay

Evans blue dye was used as a marker of albumin extravasation to evaluate alterations in retinal vascular permeability. The anesthetized rats were injected with warm Evans blue dye (2%, 45 mg/kg bw) through the jugular vein. After circulating the blood for 1 h, the eyeballs were rapidly obtained and immersed in 4% paraformaldehyde for 2 h at 25 °C in the dark. Retinas were carefully separated and unfolded on a glass slide, dried, sealed with neutral gum, and observed under a fluorescence microscope.

### 2.9 Cell culture and capsaicin treatment

Human retinal microvascular endothelial cells (HRMECs) were cultured in an endothelial cell medium (supplemented with 10% FBS and 1% penicillin-streptomycin) at 37 °C in a humidified atmosphere with 5% CO_2_. HRMECs were incubated in a 5.5 mM d-glucose medium (NG), 30 mM d-glucose medium (HG), and 30 mM d-glucose medium plus capsaicin (HG + CAP). The concentration and duration of CAP treatment were determined using the cell counting kit-8 (CCK-8) test.

### 2.10 Gene silencing

For PPARγ and poldip2 knockdown, HRMECs in the exponential growth phase were seeded into 6-well plates at 2 × 10^5^ cells/well and cultured for 24 h. The old medium was replaced with Opti-MEM before transfection. The transfection of control siRNA (100 nM) versus *poldip2* siRNA (100 nM) or control siRNA (100 nM) versus *PPARγ* siRNA (100 nM) in HRMECs was carried out using Lipofectamine 3000, in accordance with the manufacturer's instructions. After 6 h of transfection, Opti-MEM was replaced with a complete culture medium. Gene silencing was confirmed using western blotting after 72 h. The sequences of *the poldip2* siRNA (human) were 5ʹ-gcaaaguguuggagacagutt-3ʹ (forward) and 5ʹ-acugucuccaacuuugctt-3ʹ (reverse). The sequences of *PPARγ* siRNA (human) were 5ʹ-cuuaacugucggauccacatt-3ʹ (forward) and 5ʹ-uguggauccgacaguuaagtt-3ʹ (reverse). The scrambled siRNA control sequences used were 5ʹ-uucuccgaacgugucacgutt-3ʹ (forward) and 5ʹ-acgugacacguucggagaatt-3ʹ (reverse).

### 2.11 Determination of ROS

After the rats were euthanized, their eyeballs were removed rapidly. Fresh tissues were embedded in OCT, then frozen and sliced on microtome (CryoStar NX50, Thermo, Waltham, USA). Dihydroethidium (DHE) *situ* staining was performed using fresh cryosections of retinas to determine the levels of ROS and oxidative stress in the retina; the protocol was performed according to the manufacturer's instructions. The intracellular ROS concentration was measured using a ROS assay kit according to the manufacturer's protocol. The fluorescence was observed using a fluorescence microscope.

### 2.12 Detection of H_2_O_2_

Samples preparation: 5 mg retina was fully homogenized in 100 μl hydrogen-peroxide-assay lysis buffer (Beyotime, S0038), and the supernatant fluid was collected for subsequent detection. Meanwhile, whole blood was collected from the orbital vein and stored at low temperature (4 °C) for 12 h. After the clear stratification of serum and plasma, the serum was separated by centrifugation, and diluted 50 times with 50 mM phosphate buffer (pH = 6.0). The cells were collected into centrifuge tubes with hydrogen-peroxide-assay lysis buffer (100ul per 1 × 10^6^ cells). Similarity, the supernatant fluid was collected after fully homogenized. The H_2_O_2_ concentrations in the retina, serum, and cells were detected using the xylenol orange method, and the absorbance was measured at 560 nm. The assay was performed according to the instructions of the hydrogen peroxide assay kit.

### 2.13 Measurement of endothelial cell permeability *in vitro*

After 24 h of siRNA transfection, Transwell inserts were coated with 100 μl Matrigel (200–300 μg/ml) and incubated at 37 °C for 2 h. HRMECs were suspended within the serum-free medium, seeded in the upper insert chamber at a density of 2 × 10^5^/ml, and cultured at 37 °C for 1 h to allow the cells to adhere to the insert bottom. Media containing 10% FBS were added to the upper chamber (200uL) and lower chamber (1 ml), respectively. The cells were cultured in an incubator at 37 °C, 5% CO_2_ for 24 h. All Transwell inserts were transferred into a new sterile 24-well supporting plate (each well contained 1 ml fresh complete medium), the old medium in the upper chambers was removed, and 150 μl Fluorescein isothiocyanate (FITC)-dextran (10 μg/ml) was then added to the upper chambers and then incubated at room temperature for 20 min. The permeability was determined by the amount of FITC-dextran diffused into the lower wells, which was measured by the fluorescence intensity of the medium at 485 nm excitation and 535 nm emission.

### 2.14 Western blotting

At the end of treatment, the rats were euthanized. The cornea and iris of the eyeball were removed along the corneoscleral rim, and the lens was removed entirely. The vitreous humor was completely removed. The retina was bluntly separated from the sclera between the periphery and optic papilla by inserting non-toothed microforceps into the subretinal cavity. Total protein was extracted from tissues or HRMECs using RIPA lysis buffer containing protease and phosphatase inhibitors. The retinas and HRMECs were crushed using an ultrasonic cell pulverizer (scientz-IID, scientz, Ningbo, China, and the relevant set conditions are power 300W, ultrasonic 3S, interval 7S, and total time 3min) to obtain the supernatant protein, which was quantified using a BCA protein assay kit. Proteins were separated on a 10% SDS-PAGE gel and transferred to polyvinylidene fluoride (PVDF) membranes. After being blocked with 5% skim milk, the membranes were incubated with primary antibodies (anti-TRPV1, anti-PPARγ, anti-p-PPARγ, anti-ubiquitin, anti-poldip2, anti-Nox4, anti-VCAM-1, anti–HIF–1α, anti-VEGF, anti-β-actin) at 4 °C overnight, then incubated with secondary HRP-conjugated antibody for 1 h. Blots were then developed using a chemiluminescent kit according to the manufacturer's instructions.

### 2.15 Statistical analyses

Statistical analyses were performed using SPSS 22.0. All results are presented as mean ± SEM. Student's t-test was used for the analysis of two groups, and ANOVA followed by the Student-Newman-Keuls q test was used to compare multiple groups. Statistical significance was defined as *p* < 0.05. Graphs were prepared using GraphPad Prism, version 8.

## 3 Results

### 3.1 Effects of capsaicin on body weight, plasma glucose, and lipid levels of diabetic rats

**Fig. 1:**
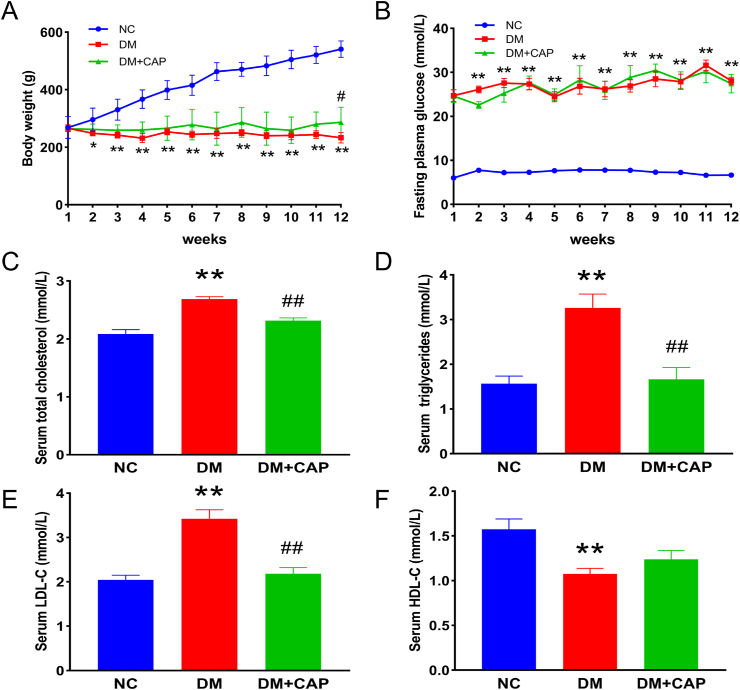
Effects of capsaicin treatment on body weights, serum glucose and lipid in rats. (A) Body weights of rats in different groups. (B) Fasting plasma glucose of rats in different groups. (C) Serum total cholesterol in rats in different groups. (D) Serum triglyceride in rats in different groups. (E) Serum low-density lipoprotein in rats in different groups. (F) Serum high-density lipoprotein in rats in different groups. One-way ANOVA, data are the means ± SEM from 5 to 8 rats/group (A, B) and 8 rats/group (C, D, E, F). Compared with NC group: *p < 0.05, **p < 0.01; Compared with DM group: ^#^*p* < 0.05, ^##^*p* < 0.01. NC: normal control group. DM: diabetic group. DM + CAP: diabetic with capsaicin treatment group.

Body weight and fasting plasma glucose levels were monitored weekly over the entire study period (12 weeks). As shown in Fig. 1A, the weight of the rats in NC group increased gradually. Compared with the NC group, the rats in DM and DM + CAP groups exhibited significantly decreased body weights. From weeks 1–11, the body weights of rats in DM and DM + CAP groups showed no significant difference. However, in week 12, compared with the DM group, the body weight of rats in the DM + CAP group showed substantial amelioration. Plasma glucose levels of rats in the NC group were in the normal range of 5.4–8.6 mmol/L, while untreated diabetes in the DM group incurred overt hyperglycemia in the range of 18.4–35.0 mmol/L, and CAP treatment did not significantly improve the hyperglycemia (Fig. 1B). Compared with those of the NC group, the TC, TG, and LDL levels of rats in the DM group were significantly increased by untreated diabetes, while CAP treatment sharply decreased these levels (Fig. 1C–E). Compared to the NC group, the HDL levels of rats in the DM group were reduced, and the CAP treatment did not significantly enhance HDL levels under diabetic conditions (Fig. 1F).

### 3.2 Capsaicin improves diabetes-induced microvessel dysfunction in the retina via regulating poldip2/Nox4 related oxidative stress *in vivo*

**Fig. 2:**
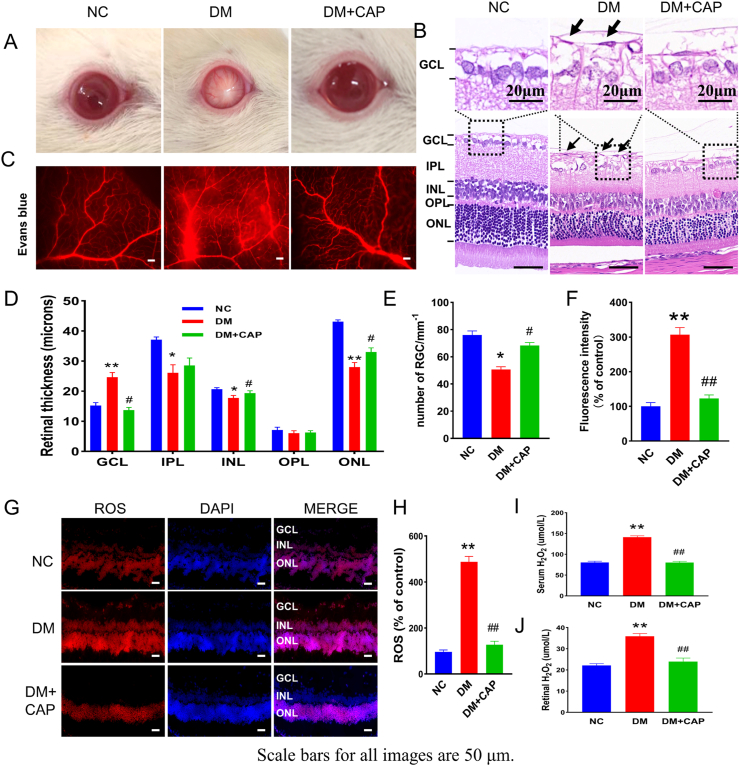
Capsaicin alleviates the pathological changes of DR and reduces ROS *in vivo*. (A) Photographs of the ocular surface of rats in different groups. (B) Photographs of HE staining of rats in different groups. (C) Photographs of Evans blue angiography of rat retina in different groups. (D) Analysis of the thickness of the subretinal layers. (E) The number of retinal ganglion cells. (F) Analysis of the fluorescence intensity. (G–H) The levels of ROS in rat retina in different groups. (I–J) The levels of H_2_O_2_ in different groups. One-way ANOVA, data represents mean ± SEM, n = 3–5/group, Scale bar: 50 μm. Compared with NC group: *p < 0.05, **p < 0.01; Compared with DM group: ^#^*p* < 0.05, ^##^*p* < 0.01. NC: normal control group. DM: diabetic group. DM + CAP: diabetic with capsaicin treatment group. (For interpretation of the references to colour in this figure legend, the reader is referred to the Web version of this article.)

As shown in Fig. 2A, with the progression of diabetes, cataracts were found in some diabetic rats. The average occurrence rate of cataracts by the end of the experiment was significantly higher in the DM group than in the control group, while the rate was significantly lower in the DM + CAP group than in the DM group (Fig. S1A and Table S1). HE staining demonstrated that the retinal ganglion cells were decreased and GCL was swollen in diabetic rats, and the inner nuclear layer (INL) and outer nuclear layer (ONL) in diabetic rats were thinner than those in healthy controls. In DM group, the inner plexiform layer (IPL) was thinner than those in the NC and DM + CAP groups, but there was no significant improvement after CAP treatment. Moreover, there was no significant difference in the thickness of the outer plexiform layer (OPL) among the groups. In addition, on the vitrea side of the ILM, preretinal neovascular cell nuclei (black arrow) were also observed in rats in the DM group. Nevertheless, after treatment with CAP, morphological changes were significantly improved in the DM + CAP group (Fig. 2B, D, E). Moreover, Evans blue angiography showed that the vascular permeability of the auricular vein in the rats in the DM group was also increased, and capsaicin treatment partially reversed this change (Fig. S1C). Retinal vascular leakage is a representative symptom of diabetic retinopathy, which was notably increased in rats in the DM group compared with those in the NC group; however, CAP treatment significantly decreased vascular leakage (Fig. 2C and F). DHE, DCFH-DA, and xylenol orange assays demonstrated an increase in oxidative stress in the retinas of diabetic rats, while CAP significantly reduced these effects (Fig. 2G–J).

**Fig. 3:**
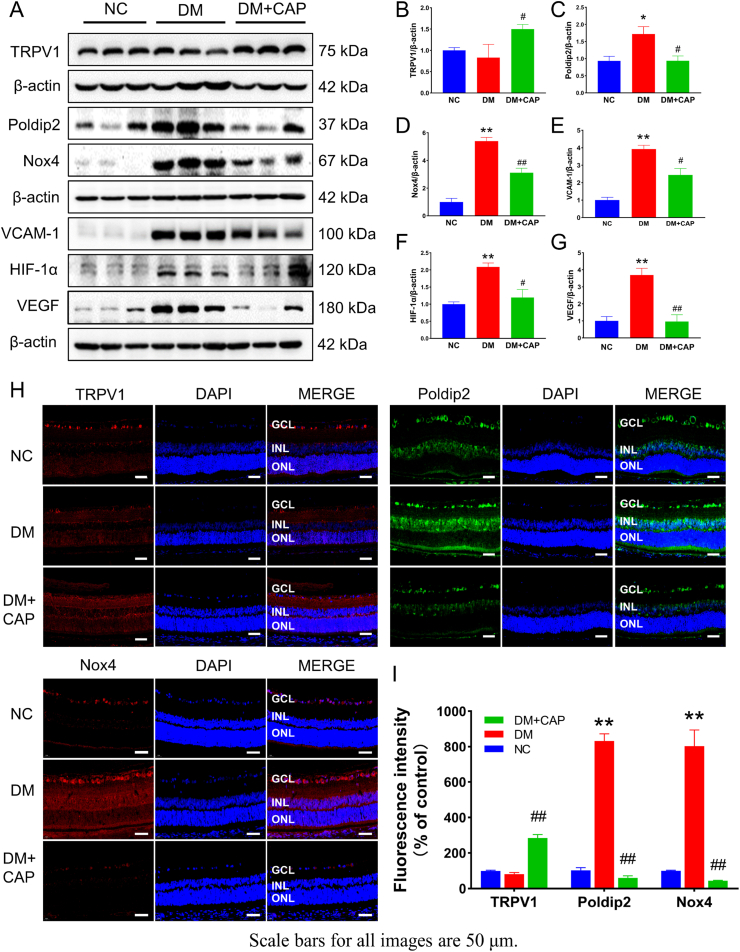
Capsaicin regulates proteins associated with oxidative stress and endothelial dysfunction *in vivo*. (A–G) Shown is the expression of TRPV1, poldip2, Nox4, VCAM-1, HIF-1α, and VEGF in rat retina in different groups. (H–I) The expression of TRPV1, poldip2, and Nox4 in rat retina by immunofluorescence in different groups. One-way ANOVA data represents mean ± SEM, n = 3–5/group, Scale bar: 50 μm. Compared with NC group: *p < 0.05, **p < 0.01; Compared with DM group: #*p* < 0.05, ##*p* < 0.01. NC: normal control group. DM: diabetic group. DM + CAP: diabetic with capsaicin treatment group.

In Fig. 3, we verified the molecular mechanism *in vivo*. DM + CAP group showed a higher TRPV1 level in the retinas of rats compared with the NC and DM groups (Fig. 3A and B). The similar result was also acquired by immunofluorescence (Fig. 3H and I). The higher luminance is equal to higher TRPV1 level. These results suggested that CAP can promote TRPV1 levels and thereby increase its activity in retinal tissues. Furthermore, the consistency between the results of western blotting and immunofluorescence for poldip2 and Nox4 supports our hypothesis. The expression of Nox4 was increased in DM group with the increase of poldip2. Furthermore, Nox4 was quickly decreased when poldip2 was markedly decreased after CAP treatment (Fig. 3A, C, D, H, I). These results fully validated previous studies on the correlation between poldip2 and Nox4. Meanwhile, VCAM-1 was increased as an inflammatory factor by ROS and hyperglycemia in DM group. It was also decreased after treatment with CAP (Fig. 3A and E). Finally, the expression of HIF-1α and VEGF was the same as other factors mentioned above, increased by hyperglycemia but decreased after CAP treatment (Fig. 3A, F, G). Our current findings demonstrated that the oxidative stress caused by diabetes could be effectively inhibited by CAP through the suppression of poldip2-Nox4 pathway.

### 3.3 Capsaicin reduces high glucose concentration-induced endothelial cell dysfunction and oxidative stress *in vitro*

**Fig. 4:**
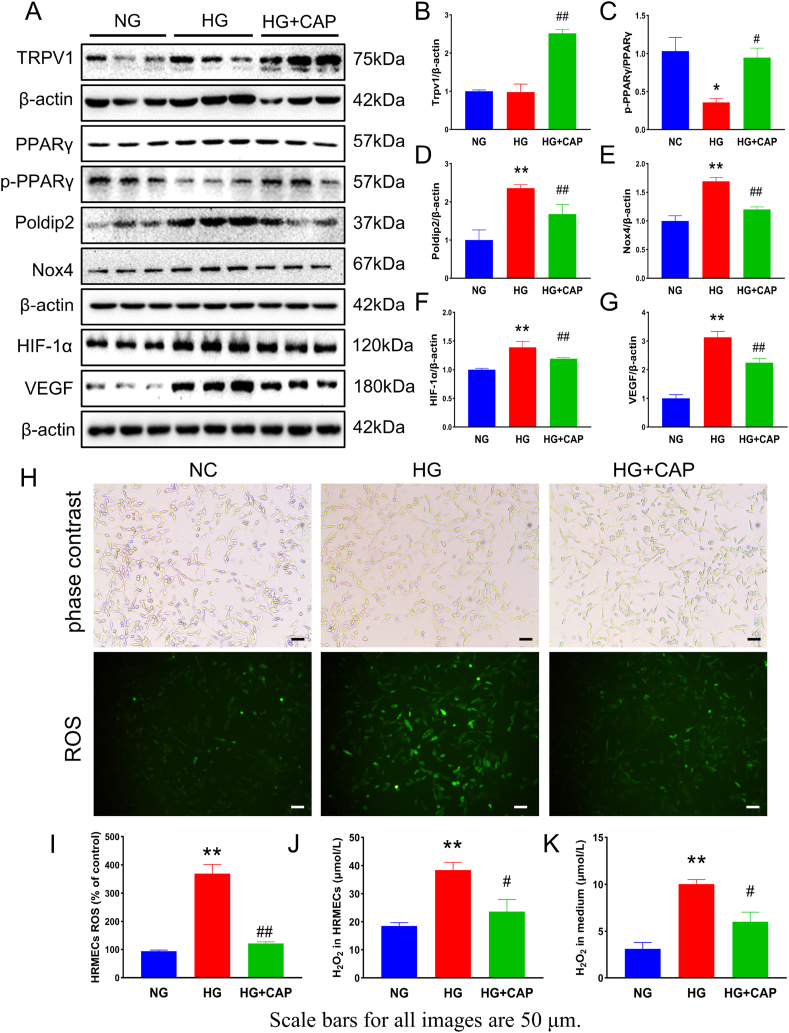
Capsaicin reduces high glucose concentration induced endothelial cell dysfunction and oxidative stress *in vitro*. (A–G) Displayed is the expression of TRPV1, PPARγ, p-PPARγ, poldip2, Nox4, VCAM-1, HIF-1α and VEGF in cultured HRMECs by different treatments. (H–I) The levels of ROS in HRMECs by different treatments. (J–K) The levels of H_2_O_2_ in HRMECs by different treatments. One-way ANOVA, data represents mean ± SEM, n = 3–5/group. Compared with NG group: *p < 0.05, **p < 0.01; compared with HG group: ^#^*p* < 0.05, ^##^*p* < 0.01. NG: normal glucose group, HG: high glucose group, HG + CAP: high glucose with capsaicin treatment group.

The optimized concentration and duration of capsaicin treatment were determined using the CCK-8 test. The concentration of capsaicin (1 μM) and treatment time (48 h) was selected for HRMECs treatment *in vitro* (Fig. S3). Moreover, the NG + CAP group was added to the *in vitro* experiments, and the target proteins (TRPV1, PPARγ, poldip2*,* Nox4, HIF-1α, VCAM-1, and VEGF) were detected in the pre-experiment. The results showed that the protein levels were not significantly different between the NG and NG + CAP groups (Fig. S2). In addition, western blotting demonstrated that the expression of TRPV1 in HRMECs of the HG + CAP group was higher than in the NG and HG groups, and there was no significant difference in TRPV1 levels between the NG and HG groups (Fig. 4A and B). Immunoblot assays confirmed an increase in PPARγ, poldip2, Nox4, VCAM-1, HIF-1α, and VEGF expression and a decrease in p-PPARγ in HRMECs exposed to high glucose concentrations. Concomitantly, CAP restored the expression of these proteins (Fig. 4A, C–G). Moreover, the bright-field photos showed that the cells were in good condition, and the number of cells in each group was the same (Fig. 4H). The additional results demonstrated increased ROS and H_2_O_2_ in cultured HRMECs and related media exposed to HG conditions, whereas CAP treatment reduced these elevations. (Fig. 4H–K).

### 3.4 Knockdown of poldip2 inhibited oxidative stress *in vitro*

**Fig. 5:**
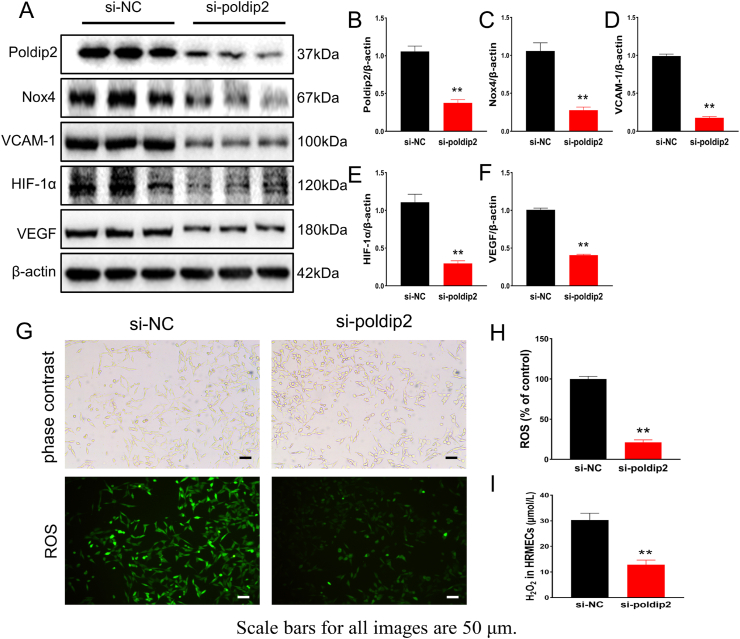
*In vitro* knockdown of poldip2 inhibited oxidative stress. (A–F) The expression of poldip2, Nox4, VCAM-1, HIF-1α and VEGF in HRMECs is demonstrated in different treatments. (G–I) Levels of ROS and H_2_O_2_ in HRMECs in different siRNA treatments. Student's t-test. Results are from three independent replicate experiments, data represents mean ± SEM, n = 3/group, scale bar: 50 μm. Compared with si-NC group: **p < 0.01. si-NC: control group, si-poldip2: *poldip2* siRNA transfection group.

To determine whether poldip2 can regulate Nox4 in HRMECs, we used small interfering RNA (siRNA) to knock down poldip2 in HRMECs. After poldip2 was silenced by siRNA in HRMECs, western blotting showed that the expression of Nox4, HIF-1α, VCAM-1, and VEGF was also significantly decreased (Fig. 5A–F). The bright-field photos showed that the cells in different groups were in good condition, with approximately the same number (Fig. 5G). DCFH-DA and xylenol orange assays showed that compared with those of the control group (si-NC), the concentrations of ROS and H_2_O_2_ were sharply decreased in the poldip2 knockdown (si-poldip2) group (Fig. 5G–I). These results suggest that oxidative stress in HRMECs can be alleviated by poldip2 knockdown.

### 3.5 Capsaicin activated TRPV1 to exert antioxidant effects through the PPARγ-poldip2*-*Nox4 pathway

**Fig. 6:**
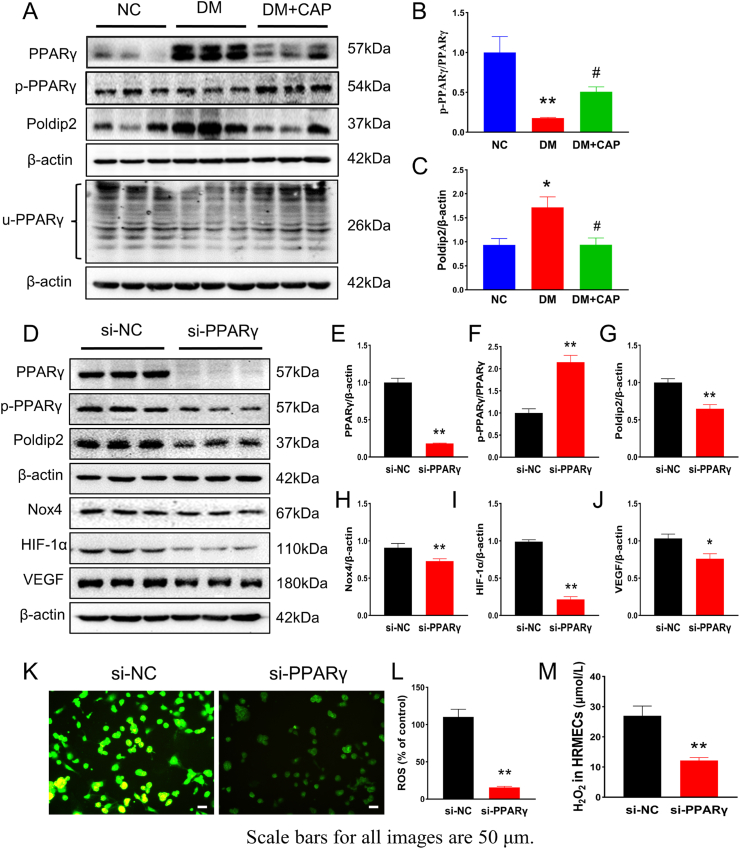
Capsaicin inhibits poldip2-mediated oxidative stress by inhibiting PPARγ. (A–C) The levels of PPARγ, p-PPARγ, u-PPARγ and poldip2 in rat retina are shown in different groups. One-way ANOVA. (D–J) Displayed is the expression of poldip2, Nox4, VCAM-1, HIF-1α and VEGF in cultured HRMECs treated by NC-siRNA and *PPARγ* siRNA, respectively. Student's t-test. (K–M) Levels of ROS and H_2_O_2_ in cultured HRMECs treated by NC-siRNA and *PPARγ* siRNA, respectively. Student's t-test. Data represents mean ± SEM, n = 3/group, scale bar: 50 μm. Compared with NG group: *p < 0.05, **p < 0.01; compared with HG group: ^#^*p* < 0.05. Compared with si-NC group: *p < 0.05. **p < 0.01. NG: normal glucose group, HG: high glucose group, HG + CAP: high glucose with capsaicin treatment group. si-NC: control group, si- PPARγ: *PPARγ* siRNA transfection group.

PPARγ plays an essential role in the pathogenesis of diabetic retinopathy (DR). Phosphorylation and ubiquitination of PPAR decrease its activity. Western blotting confirmed the increased expression of PPARγ and poldip2 and decreased p-PPARγ and u-PPARγ levels in the retinas of rats in the DM group compared with those in the NC group. CAP treatment reversed these changes (Fig. 6A–C). PPARγ was knocked down by siRNA in HRMECs to verify the relationship between PPARγ and poldip2. Western blotting showed that compared with that in the si-NC group, the expression of poldip2, Nox4, HIF-1α, and VEGF was decreased in the si-PPARγ group (Fig. 6D–J). The bright-field results showed that the number of cells in each group was similar (Fig. S4). The concentrations of ROS and H_2_O_2_ were decreased by PPARγ knockdown (Fig. 6K–M). We speculate that CAP may activate TRPV1 and exert antioxidant effects through the PPARγ-poldip2*-*Nox4 pathway.

### 3.6 Monolayer paracellular permeability was decreased by capsaicin under high glucose condition *in vitro*

**Fig. 7:**
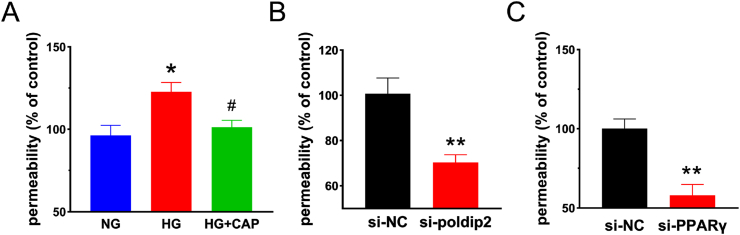
Paracellular permeability *in vitro* was detected by Transwell. (A) Exhibited is the cultured paracellular permeability in different groups with/without high glucose media. One-way ANOVA. (B) Paracellular permeability in different groups with/without silencing PPARγ in HRMECs. Student's t-test. (C) Displayed is the paracellular permeability in different groups with/without silencing poldip2 in HRMECs. Student's t-test. Data represents mean ± SEM, n = 4/group. Compared to NG group: *p < 0.05; Compared with HG group: ^#^*p* < 0.05. Compared with si-NC group: **p < 0.01. NG: normal glucose group, HG: high glucose group, HG + CAP: high glucose with capsaicin treatment group. si-NC: control group, si-poldip2: *poldip2* siRNA transfection group, si-PPARγ: *PPARγ* siRNA transfection group.

The changes in paracellular permeability of HRMECs were detected using the Transwell assay. Compared with the NG group, the leakage of FITC-dextran in the HG group was increased, revealing that the permeability of the HRMECs monolayer exposed to HG conditions was significantly increased. Compared with that of the HG group, the leakage of FITC-dextran in the HG + CAP group was decreased, indicating that paracellular permeability was decreased after capsaicin treatment (Fig. 7A). By knockdown of PPARγ in the cells, paracellular permeability was significantly reduced compared with that in the si-NC group (Fig. 7B). Similar results were observed when poldip2 was knocked down in these cells (Fig. 7C). These results indicated that CAP reduced paracellular permeability by inhibiting the expression of PPARγ and poldip2.

### 3.7 *In vivo* knockdown of poldip2 ameliorated diabetic hyperpermeability

**Fig. 8:**
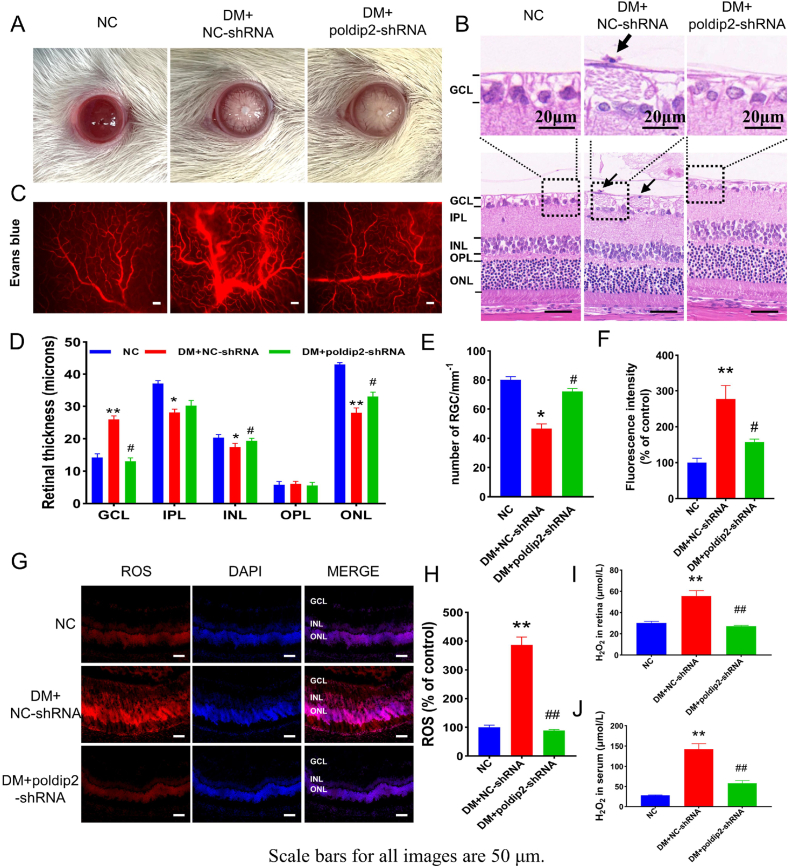
Knockdown of poldip2 *in vivo* improves the pathological changes of DR and reduces ROS. (A) Photographs of the ocular surface of rats in different groups. (B) Photographs of the HE staining of rat subretinal tissues in different groups. (C) Photographs of Evans blue angiography of rat retina in different groups with/without *poldip2*-shRNA treatment. (D) Analysis of the thickness of the rat subretinal layers. (E) The number of retinal ganglion cells. (F) Analysis of the fluorescence intensity. (G–H) The levels of ROS in rat retina in different groups with/without *poldip2*-shRNA treatment. (I–J) The levels of H_2_O_2_ in rat retina in different groups with/without *poldip2*-shRNA treatment. One-way ANOVA. Data are the means ± SEM, n = 3–4/group, scale bar: 50 μm. Compared with NC group: **p < 0.01; Compared with DM + NC-shRNA group: ^#^*p* < 0.05, ^##^*p* < 0.01. NC: normal control group. DM + NC-shRNA group: diabetic group treated with scrambled-AAV_9_-shRNA. DM + *poldip2*-shRNA: diabetic treated with AAV_9_-*poldip2*-shRNA. (For interpretation of the references to colour in this figure legend, the reader is referred to the Web version of this article.)

**Fig. 9:**
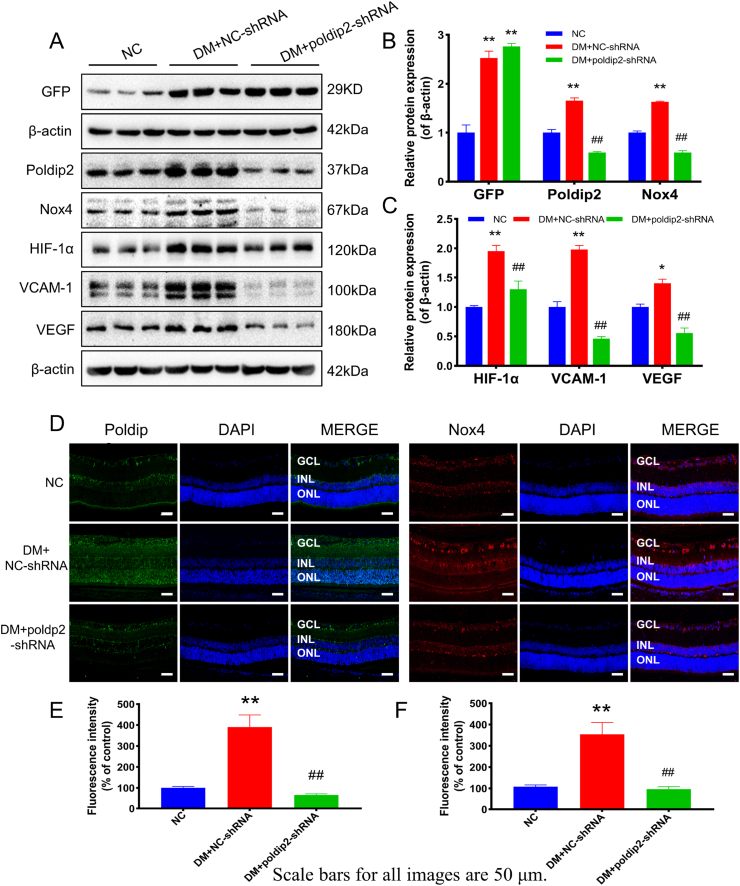
Knockdown of poldip2 *in vivo* regulates proteins associated with oxidative stress and endothelial dysfunction. (A–C) The expression of GFP, poldip2, Nox4, VCAM-1, HIF-1α and VEGF in rat retina is shown in different groups with/without *poldip2*-shRNA treatment. (D–F) The expression of poldip2 and Nox4 in rat retina detected by immunofluorescence in different groups with/without *poldip2-*shRNA treatment. One-way ANOVA. Data are the means ± SEM from 5/group (A) and 3/group (D). Compared with NC group: *p < 0.01, **p < 0.01; Compared with DM + NC-shRNA group: ^##^*p* < 0.01. NC: normal control group. DM + NC-shRNA group: diabetic group treated with scrambled-AAV9-shRNA. DM + *poldip2*-shRNA: diabetic treated with AAV_9_-*poldip2*-shRNA.

Based on the results above, poldip2 may be a major risk factor for oxidative stress in diabetic rats or cultured HRMECs under high glucose conditions. Therefore, we used AAV_9_ vectors carrying an shRNA against poldip2 to downregulate the expression of poldip2 in the retinal vasculature through intravitreal injection in the eyes of diabetic rats. Cataracts were found in diabetic rats (Fig. 8A). Compared with the DM + NC-shRNA group, fewer cataracts were observed in the DM + *poldip2*-shRNA group (Fig. S1A and Table S1). HE staining results showed that the structure of each layer of the retina in the NC group was clear, and that the cells in the inner nuclear layers (INL) and outer nuclear layers (ONL) are closely arranged. In the DM + NC-shRNA group, the number of ganglion cells was decreased, and the INL and ONL became thinner compared with the normal control group. After the knockdown of poldip2, the pathological changes in the diabetic rat retina were significantly improved. The thickness of the OPL among the groups were no significant difference. (Fig. 8B, D, E). In addition, the results of Evans blue angiography showed that the vascular permeability of the auricular vein in the DM + NC-shRNA group was also increased and that in the DM + *poldip2*-shRNA group; the infiltration of Evans blue in the auricular vein was significantly reduced (Fig. S1D). Although retinal vascular leakage was notably increased in the DM + NC-shRNA group compared with the NC group, the retinal vascular leakage in the DM + *poldip2*-shRNA group was significantly decreased (Fig. 8C, F). The levels of ROS and H_2_O_2_ in retinal tissue and H_2_O_2_ in serum were increased in the DM + NC-shRNA group, while oxidative stress was significantly reduced in the DM + *poldip2*-shRNA group after poldip2 knockdown *in vivo* (Fig. 8G–J). The effect of AAV_9_-*poldip2*-shRNA knockdown on the retinal vasculature was confirmed by western blotting. Compared with the NC group, the expression of GFP protein was significantly increased in the DM + NC-shRNA and DM + *poldip2*-shRNA groups (Fig. 9A). Immunoblots demonstrated that the expression of poldip2, Nox4, VCAM-1, HIF-1α, and VEGF was sharply decreased in the retinas of rats in the DM + *poldip2*-shRNA group compared with the DM + NC-shRNA group (Fig. 9A–C). The expression of poldip2 and Nox4 by IHF assays was consistent with that observed by immunoblotting (Fig. 9D–F).

### 3.8 Capsaicin reduces inflammation by inhibiting poldip2

*In vivo* immunoblots showed that compared with the NC group, the expression of VCAM-1 in the retinas of rats was increased in the DM group, and CAP reversed this change (Fig. 3A). The expression of VCAM-1 in the DM + NC-shRNA group was higher than that in the NC group. After the knockdown of poldip2 *in vivo*, the expression of VCAM-1 decreased (Fig. 9A). *In vitro*, compared with that in the si-NC group, VCAM-1 expression in the si-poldip2 group was decreased with the reduction in poldip2 (Fig. 5A).

## 4 Discussion

It has been well documented that chronic hyperglycemia in diabetes increases the polyol and hexosamine pathways, accumulation of advanced glycation end products (AGEs), and activation of protein kinase C (PKC), all of which can lead to oxidative stress and the occurrence of DR through endothelial cell dysfunction and new angiogenesis [3].

Our current results suggest that poldip2 is highly expressed in the high-glucose state, which produces excessive H_2_O_2_ by activating Nox4. Excessive ROS (H_2_O_2_) can cause the overexpression of VCAM-1, HIF-1α, and VEGF. In addition, high levels of poldip2 can also promote the expression of VCAM-1, causing inflammation and resulting in the progression of DR. Based on our current data, we found that CAP may inhibit oxidative stress and reduce vascular permeability and neovascularization. This may be due to the CAP-induced activation of TRPV1 and its mediated PPARγ-poldip2-Nox4 signaling pathway, thereby inhibiting the progression of DR.

CAP is an agonist of TRPV1, a transmembrane channel that prefers Ca^2+^. In addition to being expressed in sensory neurons, TRPV1 is also expressed in the vascular endothelial cells. In this study, immunofluorescence assay of rat retina and immunoblots of retinal tissue and HRMECs confirmed that compared with the NC and DM groups, the expression of TRPV1 in the capsaicin-treated group was significantly increased, indicating the effective activation of TRPV1 in retinal tissues and cultured cells.

Nox4 and Nox2 expression was increased in the retinas of a streptozotocin-induced diabetic rat model, which was closely associated with increased levels of oxidative stress in the retina [21]. Based on our current data, in the state of hyperglycemia, the expression of poldip2, Nox4, and H_2_O_2_ increased, whereas CAP reduced oxidative stress and endothelial damage by inhibiting the levels of *polidp2*, Nox4, and H_2_O_2_. H_2_O_2_ can also promote hypoxia-inducible factor-1α (HIF-1α) levels and increase angiogenesis in hypoxic tissue. Our current data also showed that excessive H_2_O_2_ induced HIF-1α gene overexpression and that HIF-1α protein elicited increased expression of VEGF in the retinas of rats with DR and HRMECs cultured in high glucose media. A published study showed that retinal ischemia and hypoxia during DR can induce increased expression of HIF-1α, which promotes the expression of vascular endothelial growth factor (VEGF) [22]. Studies have shown that diabetic retinopathy can be improved by inhibiting HIFs-VEGF signaling [[23], [24], [25]]. These results were consistent with the current results. In addition, our current study found that CAP treatment reversed these changes. In addition, a previous study showed that poldip2 could promote the expression of VCAM-1, causing inflammation in rat brain microvascular endothelial cells [10]. Hyperglycemia and H_2_O_2_ can also increase the expression of VCAM-1 [9,26]. Similarly, our current study found that the levels of VCAM-1 in the retinas of rats with DR and HRMECs cultured in high glucose were increased and decreased after CAP treatment. Moreover, after the knockdown of poldip2 *in vitro* and *in vivo*, the levels of ROS, H_2_O_2_ sharply decreased, along with a drastic reduction in the expression of VCAM-1, HIF-1α, and VEGF. *In vivo*, the retinal structure was improved, and neovascularization and retinal microvascular leakages were decreased after knockdown of poldip2. Silencing of the poldip2 gene in HRMECs also reduced cell permeability. In addition, research has shown that poldip2 deficiency curbs VCAM-1 induction and mitochondrial ROS production induced by LPS *in vitro* [12]. Knockdown of the poldip2 gene can improve blood-brain barrier (BBB) permeability [11]. These results were consistent with the current data.

Oxidative stress can cause microvascular dysfunction and barrier damage, leading to retinal microangiopathy [3]. VEGF can promote vascular leakage and angiogenesis, a manifestation of DR pathogenesis. The increased expression of VCAM-1 boosts leukocyte adhesion and induces endothelial dysfunction [10]. In the current study, HE staining showed that the structure of diabetic retinal was disordered and that neovascularization occurred in the untreated DM group. Evans blue staining revealed that, compared with the NC group, retinal microvascular leakage was obviously enhanced in DR rats. However, CAP treatment improved retinal structure and reduced retinal neovascularization and vascular leakage. Moreover, the Transwell assay *in vitro* showed that the paracellular permeability of the HRMECs monolayer cultured in high-glucose media was significantly higher than that of the control group, and that the cellular hyperpermeability was greatly improved after capsaicin treatment. A recent study showed that capsaicin can increase the level of UCP2, both in porcine iliac artery endothelial cells (PIECs) cultured in a high glucose medium and in db/db mice, and alleviate ROS production and endothelial dysfunction induced by high glucose or hyperglycemia [27]. These results are consistent with our results.

To explore the mechanism of capsaicin-mediated downregulation of poldip2 in the pathological state of DR, we conducted further studies. PPARγ has been found to be involved in this process. The phosphorylation and ubiquitination of PPARγ can significantly suppress its activity or lead to its degradation [28]. Our current results showed that, compared with the NC group, the expression of PPARγ and poldip2 was increased, while the levels of p-PPARγ and u-PPARγ were decreased in the retinal tissues of DR rats. However, CAP treatment reversed these changes. Similar results were obtained *in vitro*. It has been documented that in bovine retinal vascular endothelial cells, hyperglycemia increases the expression of PPARγ1, PPARγ2 mRNA, and PPARγ protein, and that the expression of the retinal protein PPARγ in diabetic rats is significantly increased [16]. In addition, CAP efficiently suppresses adipogenesis in 3T3-L1 preadipocytes and adipocytes by downregulating PPARγ expression [17]. These results are consistent with our study's data. One study showed that the antioxidant capacity of PPARγ ± heterozygous mice is enhanced [29]. Furthermore, in the current study, after knockdown of PPARγ in HRMECs, the expression of poldip2, Nox4, VCAM-1, VEGF, and levels of ROS and H_2_O_2_ were evidently decreased, and the paracellular permeability of the HRMECs monolayer in the PPARγ-silenced group was reduced. To date, no published studies have reported the effect of PPARγ on poldip2, except our current research, which indicated that PPARγ closely regulates poldip2 expression, and that in the retina of DR rats and in HRMECs cultured in high glucose, CAP can inhibit the poldip2-Nox4 signaling pathway by downregulating the expression of PPARγ, thus inhibiting oxidative stress and inflammation, and delaying the occurrence and development of DR.

PPARγ also regulates several metabolic diseases such as obesity and diabetes [30]. However, for a long time, whether to activate or inhibit PPARγ activity to exert insulin sensitization has been a controversial issue. Thiazolidinediones (TZDs), PPAR agonists, are the only class of FDA-approved insulin sensitizers. It is used for the treatment of diabetes with a definite effect. However, many adverse reactions have hindered the continued use of these drugs. In addition, one published study showed that compared with wild-type mice, PPARγ ± heterozygous mice showed lower expression of PPARγ, but higher insulin sensitivity [31]. Furthermore, PPARγ deficiency in PPARγ ^±^ heterozygous mice leads to increased expression of ROS-scavenging genes, thus enhancing tolerance to oxidative stress [29]. Therefore, we speculated that PPARγ-mediated insulin sensitization may depend on the basic expression level and activity of PPARγ. Adjusting the activity of PPARγ to an appropriate level may be the basis of its effect.

One study has shown that CAP can reduce blood glucose by improving glucose metabolism disorders [32]. However, in this study, CAP intervention did not significantly improve blood glucose levels in diabetic rats, which may be related to the lower concentration of CAP or the shorter intervention time. Diabetes is a wasting disease that can lead to weight loss, and a study on CAP demonstrated the effect of weight loss [33]. Our results showed that compared with the NC group, the body weight of the rats in the DM group decreased significantly. However, the body weight of the rats in the DM + CAP group showed no significant decrease compared with the DM group, and even increased in the eighth week post CAP treatment. We speculate that there are two main reasons for this discrepancy. First, compared to obese rats or mice, the type 1 diabetes model used in this study had less fat, and the lipid-lowering effect of CAP was limited. Second, it may be related to the fact that diabetes-associated symptoms were significantly improved, thus reducing energy expenditure in diabetic rats after CAP treatment.

**Fig. 10:**
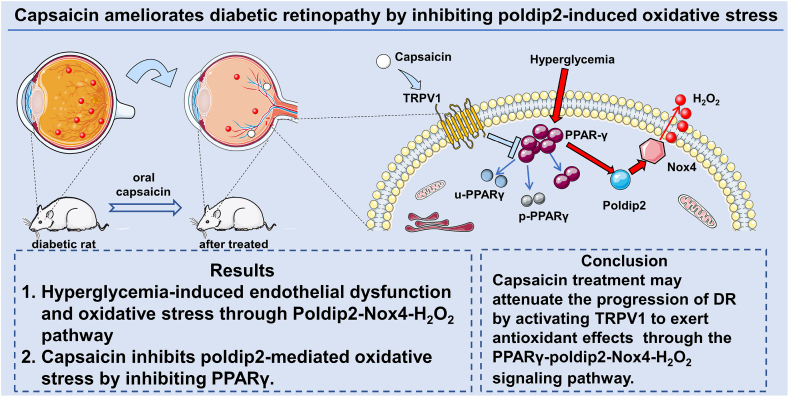
Schematic diagram of this study. Hyperglycemia-induced endothelial dysfunction and oxidative stress through poldip2-Nox4-H_2_O_2_ pathway, while capsaicin may ameliorate diabetic retinopathy by activation of TRPV1 and then suppression of PPARγ-poldip2-Nox4 pathway.

As shown in the graphical abstract (Fig. 10), we found that CAP decreased the concentration of peroxide. The mechanism may be that CAP can activate TRPV1 receptor, which enhances the phosphorylation and then ubiquitination of PPARγ. As a result, the activity of PPARγ increased by hyperglycemia is inhibited, and the poldip2 is downregulated as its upstream transcription factor (PPARγ) has been inhibited. Finally, the progression of poldip2-Nox4-H_2_O_2_ singling is suppressed, and oxidative stress is thus alleviated.

## 5 Conclusions

In this study, we found that CAP treatment may attenuate the progression of DR by reducing oxidative stress and the inflammatory response, or by inhibiting retinal angiogenesis and suppressing vascular leakage through the PPARγ-poldip2-Nox4-H_2_O_2_ signaling pathway. Our findings suggest that capsaicin is a potential novel agent for the treatment of DR in clinical practice.

## Ethics approval and consent to participate

The study was approved by the Institutional Animal Care and Use Committee of Anhui Medical University and was performed in accordance with relevant guidelines and regulations.

## Funding

This study was supported by the 10.13039/501100001809National Natural Science Foundation of China (Nos.82070986 and 82171043).

## Author contributions

Zhengxuan Jiang and Liming Tao designed the study, provided financial support, and revised the manuscript accordingly. Kou Liu, Xiang Gao, Yanchao Gui, Siyu Gui, and Qinyu Ni performed all experiments. Liu, Gao, and Hu analyzed the data. Kou Liu and Xiang Gao drafted the manuscript. All authors have read and approved the final manuscript.

## Declaration of competing interest

The authors declare that they have no conflict of interest.

## Data Availability

Data will be made available on request.
