# Supplementary material for: Capsaicin ameliorates diabetic retinopathy by inhibiting poldip2-induced oxidative stress
Source: Redox Biol. 2022 Sep 3;56:102460. doi: 10.1016/j.redox.2022.102460 (PMC9468458; doi:10.1016/j.redox.2022.102460)
Supplement: Multimedia component 1 [file mmc1.docx]

**Supplementary data**

**Figure legends**

**Fig. S1** (A) Photographs of the ocular surface of rats in different groups with /without capsaicin treatment. (B) Photographs of the ocular surface of rats in different groups with /without *poldip2*-shRNA treatment. (C) Photographs of marginal auricular vein of rat retina in different groups. (D) Photographs of marginal auricular vein in different groups with /without *poldip2*-shRNA treatment. NC: normal control group. DM: diabetic group. DM+CAP: diabetic with capsaicin treatment group. DM+NC-shRNA group: diabetic group treated with scrambled-AAV_9_-shRNA. DM+ *poldip2*-shRNA: diabetic treated with AAV_9_-*poldip2*-shRNA.

**Fig. S2** Shown is the expression of TRPV1, poldip2, Nox4, VCAM-1, HIF-1α, and VEGF in HRMECs in NG, NC+CAP, HG and HG+CAP groups. NG: normal glucose group, NG+CAP: normal glucose with capsaicin treatment group, HG: high glucose group, HG+CAP: high glucose with capsaicin treatment group.

**Fig. S3** Determination of cell viability. Optimal time and concentration of capsaicin treatment were determined based on the cell viability by CCK8 test, including concentration gradient (0, 0.5, 1.5, 2 μM), treatment times (0, 24, 48, 72 h). Data were shown as mean ± SEM for each experiment performed independently 3 times.

**Fig. S4** The levels of ROS in HRMECs in different groups with /without *PPARγ* siRNA treatment. si-NC: control group, si-PPARγ: PPARγ siRNA transfection group.

**Table S1** Effect of capsaicin or AAV_9_-*poldip2*-shRNA on incidence of cataract in STZ-induced diabetic rats. NC: normal control group. DM: diabetic group. DM+CAP: diabetic with capsaicin treatment group. DM+NC-shRNA group: diabetic group treated with scrambled-AAV_9_-shRNA. DM+ *poldip2*-shRNA: diabetic treated with AAV_9_-*poldip2*-shRNA.


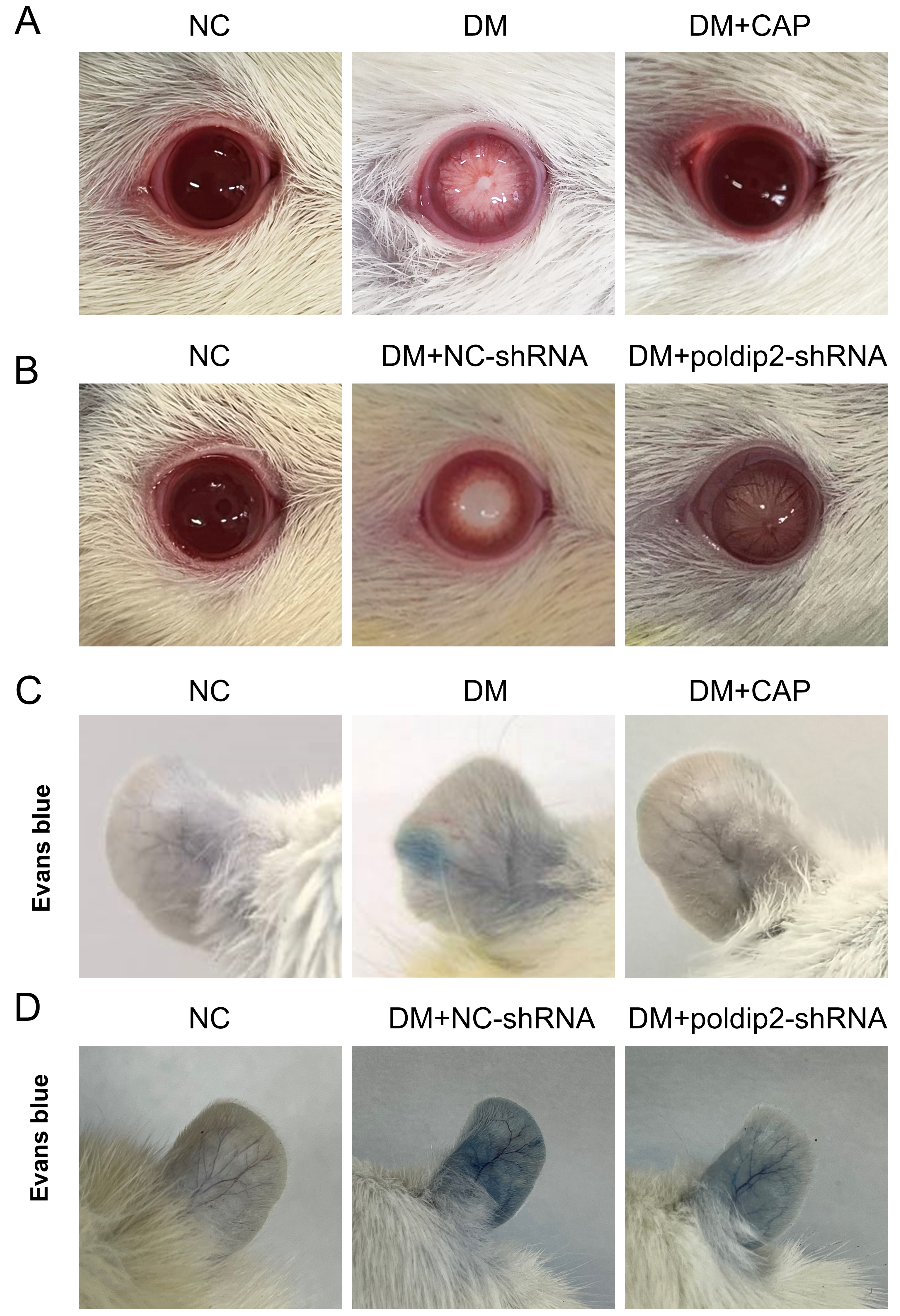


**Fig. S1**


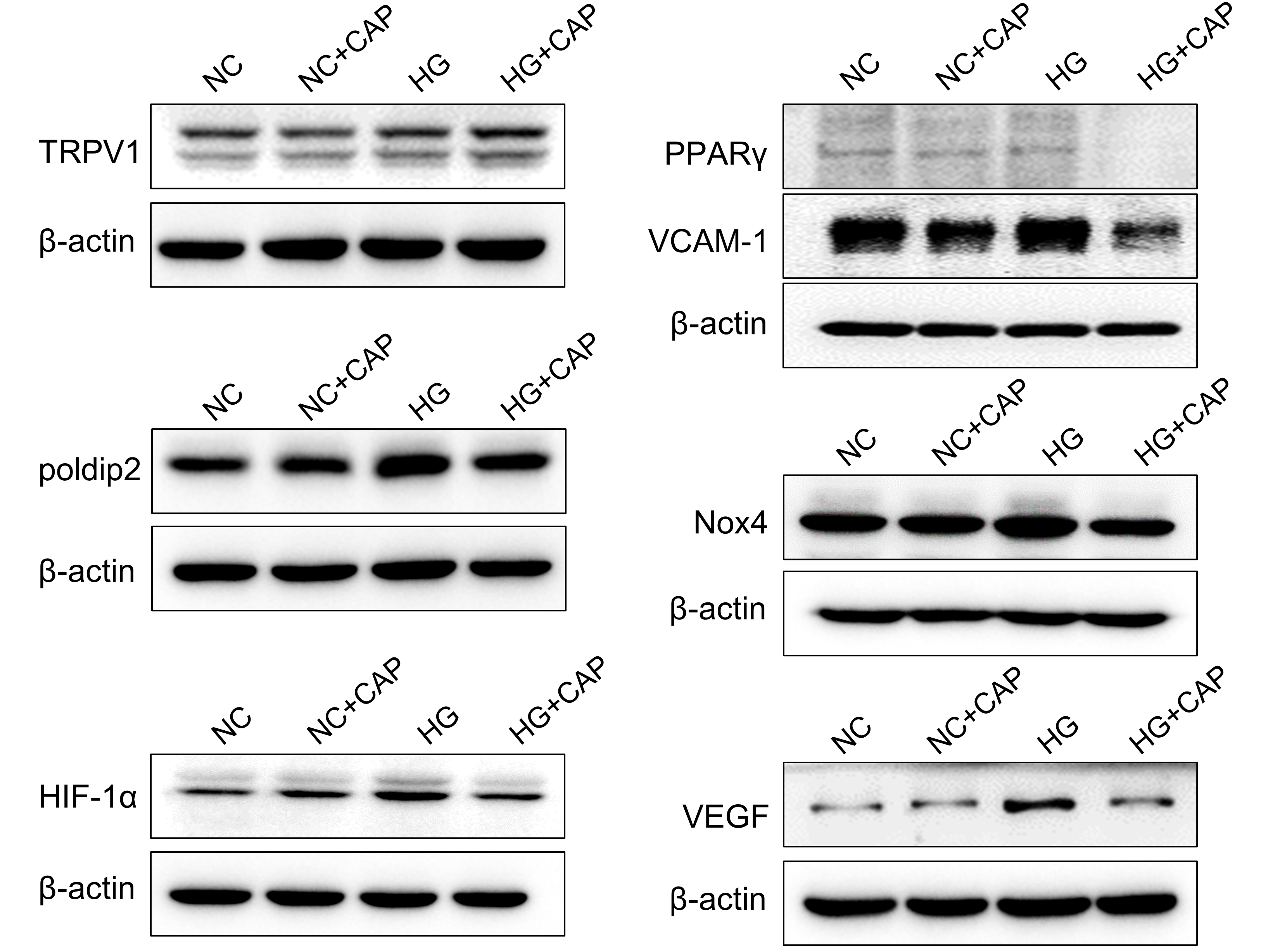


**Fig. S2**


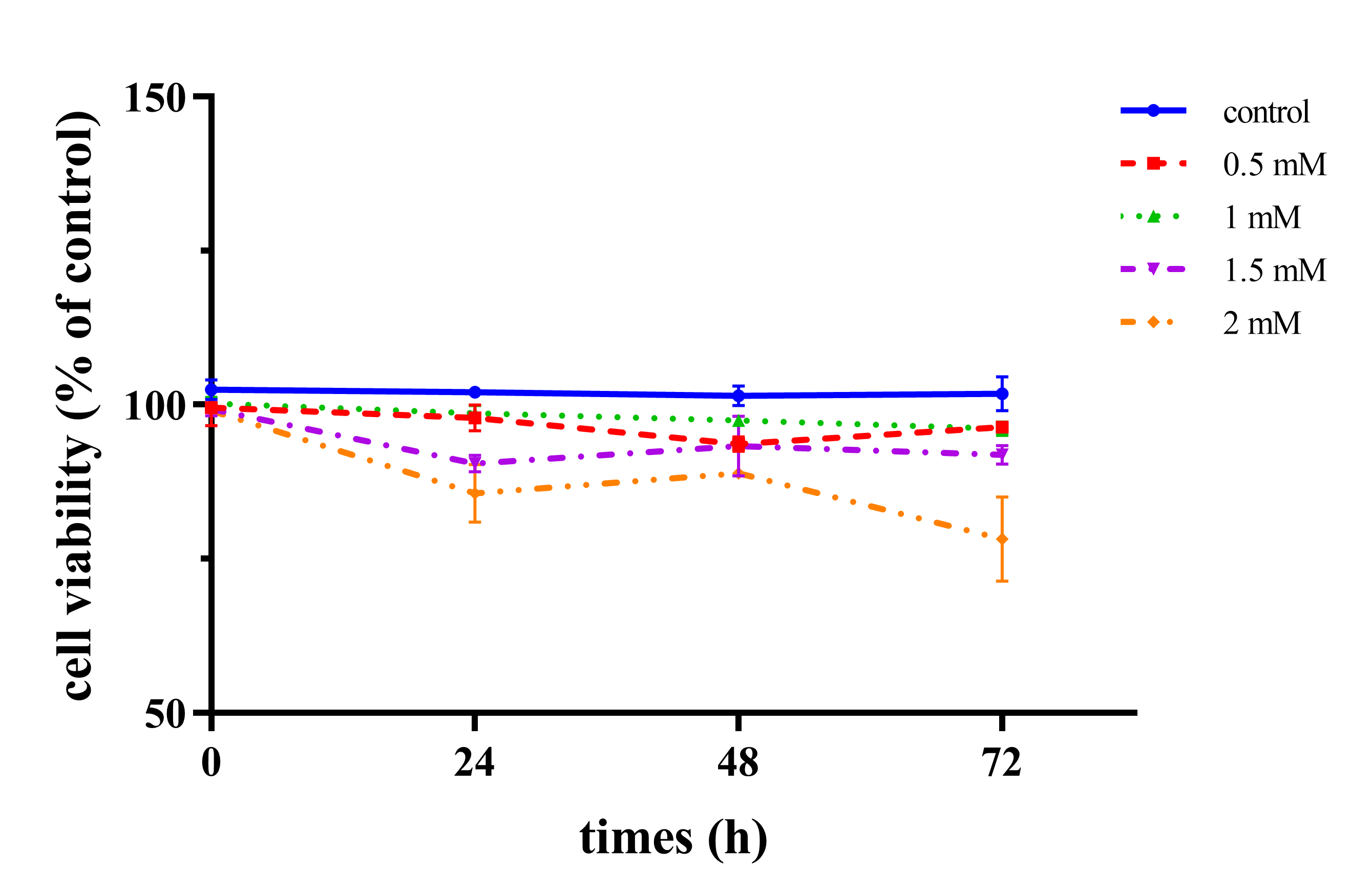


**Fig. S3**


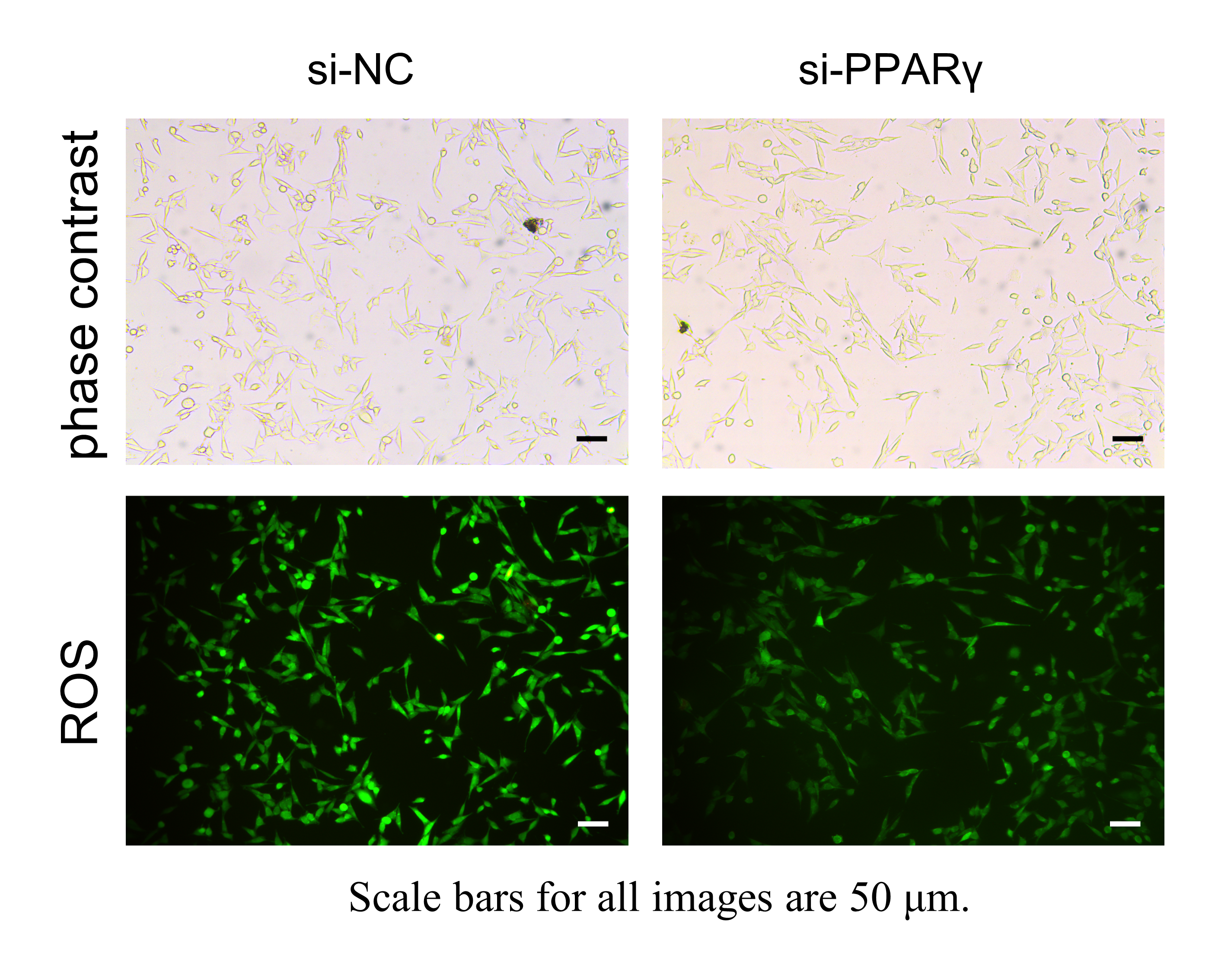


**Fig. S4**

**Table S1** Effect of capsaicin or AAV_9_-*poldip2*-shRNA on incidence of cataract in STZ-induced diabetic rats.

| Groups | Total eyes | Cataract incidence  (Number of eyes with cataract) |
| --- | --- | --- |
| NC | 20 | 0 |
| DM | 20 | 19 |
| DM+CAP | 20 | 14 |
| DM+NC-shRNA | 20 | 16 |
| DM+*poldip2*-shRNA | 20 | 12 |
